# Psychiatric Diagnosis at Prison Admission: Clinical and Sociodemographic Characteristics of Newly Admitted Inmates

**DOI:** 10.3390/healthcare14213682

**Published:** 2026-10-30

**Authors:** Raffaele Gaetano, Elvira Anna Carbone, Vincenzo Pitone, Martina Sacco, Renato de Filippis, Valentina Pugliese, Francesca Bruno, Pasquale De Fazio, Giulio Di Mizio

**Affiliations:** 1 Prison Medical Health Center, Intensive Assistance Service (SAI), Catanzaro Health Authority “U. Caridi” Correctional Center (Italian Ministry of Justice), 88100 Catanzaro, Italygiulio.dimizio@unicz.it (G.D.M.); 2 Department of Health Sciences, “Magna Graecia” University of Catanzaro, 88100 Catanzaro, Italy; 3 Forensic Medicine and Criminology, Law Department, University Campus “S. Venuta”, 88100 Catanzaro, Italy

**Keywords:** prisons, prison admission, psychiatric disorders, substance use, correctional psychiatry, forensic psychiatry

## Abstract

**Highlights:**

**What are the main findings?**

• Around 41% of newly admitted inmates had a psychiatric diagnosis at prison entry.
• Substance use was the strongest factor associated with psychiatric morbidity on admission.

**What are the implications of the main findings?**

• Prison admission is a key opportunity for psychiatric screening.
• Integrated care for mental disorders and substance use is needed.

**Abstract:**

**Background/Objectives**: Individuals entering prison represent a highly vulnerable population with substantial mental health needs. While the prevalence of psychiatric disorders in correctional settings has been widely documented, evidence on the factors associated with a psychiatric diagnosis at prison admission remains limited. This study aimed to characterize the clinical and sociodemographic profile of newly admitted inmates and to identify independent factors associated with a psychiatric morbidity at prison entry. **Methods**: This retrospective cross-sectional study included individuals newly admitted to the Correctional Center in Southern Italy between 2023 and 2025. Routinely collected sociodemographic, legal, and clinical admission data were analyzed. Participants with and without psychiatric diagnosis at admission were compared and independent factors associated with diagnosis at admission were identified through regression analyses. **Results**: Among 707 newly admitted male inmates, ~41% had a documented psychiatric diagnosis at prison entry, were younger, more frequently of foreign nationality, and more likely to report substance use. In multivariable analysis, substance-use profiles—particularly combined use of cannabis, cocaine, and psychostimulants (aOR = 11.55, *p* < 0.001)—and foreign nationality (aOR = 4.29, *p* < 0.001) were independently associated with documented psychiatric morbidity at prison admission. Awaiting final judicial disposition, age and first incarceration showed no significant association. **Conclusions**: A substantial proportion of individuals entering prison present with a psychiatric morbidity, with specific substance use profiles showing a strong independent association with outcome. These findings highlight the clinical complexity of individuals entering correctional settings and support systematic psychiatric assessment.

## 1. Introduction

Mental disorders among the incarcerated population represent a major public health concern. According to the World Health Organization (WHO), the global prison population exceeds eleven million people, and approximately one third of this population is estimated to be affected by at least one mental disorder [1,2]. Psychiatric disorders are consistently more prevalent in the prison population than in the general population, with similar patterns reported across different geographical regions, including Europe and North America [1,2,3]. Recent evidence confirmed the substantial burden of severe mental illness in prison populations. A systematic review and meta-analysis including approximately 60,000 individuals from 43 countries estimated a pooled prevalence of 12.8% for depression, 4.1% for psychotic disorders, 3.6% for schizophrenia spectrum disorders, and 1.7% for bipolar disorders [4]. Other evidence confirmed that around half of the prison population have a comorbid substance use disorder [5] and approximately one out of every four adults in custody have a dual diagnosis [6]. These findings confirm that severe mental illnesses are markedly overrepresented among incarcerated populations compared with the general population.

Previous Italian studies reported high rates of psychiatric disorders and comorbidity, particularly involving substance use disorders [7,8,9,10], and highlighted the relevance of dual diagnosis as a central issue in correctional mental healthcare [7,11]. Beyond the prevalence of single psychiatric disorders, incarcerated populations are characterized by high levels of psychiatric comorbidity. The coexistence of mental disorders and substance use disorders is particularly relevant [12] as it is associated with greater clinical severity, increased risk of suicide, poorer adherence to treatment, and a higher likelihood of criminal recidivism after release [4,13,14]. Therefore, early identification of psychiatric morbidity is essential to provide timely interventions and ensure continuity of care within correctional healthcare systems [15,16].

Despite the recognized burden of psychiatric morbidity in prisons, several important knowledge gaps remain. First, most Italian studies [7,9,14] have focused on the prevalence of psychiatric disorders among incarcerated populations or on the implementation of mental health screening programs. Recent Italian experiences implementing structured psychological screening at prison entry have highlighted the feasibility and clinical relevance of early assessment strategies [14]. However, evidence regarding the independent sociodemographic, legal and clinical factors associated with a psychiatric diagnosis at prison admission remains limited. Identifying readily available factors associated with psychiatric morbidity may allow for the development of targeted screening strategies and more efficient allocation of mental health resources within correctional settings. Although substance use disorders are highly prevalent among inmates, the contribution of specific substance use patterns to psychiatric morbidity at prison entry remains insufficiently explored.

Finally, evidence from Southern Italy remains scarce, despite relevant differences in healthcare organization, socioeconomic context, and availability of specialized services compared with other geographical areas. Moreover, few studies have comprehensively described the sociodemographic, legal, and clinical characteristics of individuals at prison admission over a defined observation period.

Therefore, this study aimed to characterize the sociodemographic, legal, and clinical profile of individuals newly admitted to a correctional facility in Southern Italy between 2023 and 2025 and to identify independent factors associate with a psychiatric morbidity at prison admission. Characterizing these associations may help correctional healthcare services anticipate the distribution of mental health needs at admission, identify groups requiring particular clinical attention, and inform the organization of integrated psychiatric and substance-use assessment. By focusing on the prison entry phase, this study seeks to provide evidence to support risk-based psychiatric screening and integrated mental health care within Italian correctional services.

## 2. Materials and Methods

### 2.1. Study Design and Aims

This study was designed as a retrospective observational cross-sectional analytical study aimed at describing the sociodemographic, legal, and clinical characteristics of newly inmates admitted to the prison healthcare service “U. Caridi” Correctional Center in Catanzaro, Southern Italy, between March 2023 and December 2025.

The primary objective was to characterize the study population according to sociodemographic, legal, and clinical variables.

Secondary objectives were to:

(i) Compare individuals with and without a psychiatric diagnosis at admission according to the main sociodemographic, legal, and clinical characteristics;
(ii) Identify independent factors associated with a psychiatric diagnosis at admission through multivariate logistic regression analysis.

### 2.2. Participants

The study included all consecutive individuals newly admitted to the prison facility during the study period. Individuals were included only at their first admission during the observation period to avoid duplicate observations. Data were collected from clinical and administrative records of inmates evaluated by the prison healthcare service “U. Caridi” Correctional Center during the study period. The following variables were extracted:

• Sociodemographic characteristics (e.g., age and nationality);
• Legal status at admission (e.g., awaiting final judicial disposition/sentenced and first incarceration experience);
• Clinical characteristics (e.g., substance use, documented psychiatric history, current psychiatric diagnosis and ongoing pharmacological treatment).

Documented psychiatric diagnosis at admission was defined as the presence of psychiatric morbidity documented in the available clinical documentation and/or identified during the psychiatric assessment performed by expert psychiatrists at admission.

### 2.3. Data Collection and Management

Clinical and administrative data were reviewed retrospectively and collected in a dedicated anonymized database to ensure confidentiality and protection of personal data. Administrative data were used for variables routinely and systematically recorded as part of the institutional admission process. These variables were obtained from the correctional facility’s official administrative records and were not derived from patient self-report for the purposes of this study. Clinical variables were obtained from the clinical documentation and psychiatric assessments available within the correctional healthcare service. Psychiatric diagnoses—according to the DSM-5 [17]—were established by psychiatrists according to routine clinical practice, supplemented by available clinical documentation, during the admission evaluation. The study was conducted in accordance with the principles of the Declaration of Helsinki. As this was a retrospective study based on anonymized clinical data collected during routine healthcare activities, formal ethical approval was waived according to local regulations.

### 2.4. Statistical Analysis

Statistical analyses were performed using JASP statistical software (version 0.18.3; JASP Team, Amsterdam, The Netherlands, 2024). Continuous variables were summarized using mean (M) and standard deviation (SD). Categorical variables were reported as absolute frequencies and percentages. The total sample was stratified into two cohorts based on the presence or absence of a psychiatric morbidity on admission.

First, a descriptive and bivariate analysis was conducted to explore differences in baseline sociodemographic, legal, and clinical characteristics between the two groups. Continuous variables were expressed as M ± SD and analyzed using the independent samples *t*-test; Cohen’s d was computed to evaluate the corresponding effect size. Categorical variables were summarized as frequencies (Fr) and percentages (%), and statistical associations were assessed using Chi-squared (χ^2^) test. For categorical comparisons, effect sizes were quantified using Cramer’s V coefficient. Fisher’s exact test was cross-verified for 2 × 2 contingency tables presenting low expected frequencies or zero-cell counts.

To identify independent factors associated with a psychiatric diagnosis at admission, a multivariate logistic regression model was used. Variables included in the model were selected based on clinical relevance and/or significant associations observed in preliminary analyses. To prevent oversimplification and capture the nuanced clinical impact of substance use profiles, the generic dichotomous substance use variable was replaced in the multivariant model with its specific categorical subtypes (non-users, cannabis only, opioids only, cannabis + cocaine + psychostimulants, and cannabis + opioids), using non-substance users as the reference group. All other relevant sociodemographic and legal covariates (age, nationality, first incarceration status, and legal status) were entered simultaneously into the model to control for mutual confounding.

Results from logistic regression were reported as adjusted odds ratios (aORs) with corresponding 95% confidence intervals (CIs) and *p*-values. Model performance was evaluated using the likelihood ratio test and pseudo-R^2^ indices, including McFadden’s, Nagelkerke’s, Tjur’s, and Cox & Snell’s R^2^ values. Nationality was dichotomized as Italian versus foreign, and age was entered as a continuous variable; the linearity assumption between age and the logit of the outcome was assessed and no relevant departure from linearity was identified.

Additionally, a sensitivity analysis was performed using Firth penalized logistic regression to assess the robustness of the regression estimates. The same outcome, covariates, and reference categories used in the primary logistic regression model were retained. For all analyses, statistical significance was set a priori at *p* ≤ 0.05.

## 3. Results

### 3.1. Sample Description

A total of 707 male participants were included in the study. The main demographic, legal, and clinical characteristics of the sample are summarized in Table 1. The mean age of the participants was 42.7 ± 13.0 years. Most participants were of Italian nationality (89.8%), whereas smaller proportions originated from the Maghreb region (3.7%), other European countries (3.4%), or other geographic areas (3.1%).

Regarding legal status, 62.8% of the sample were awaiting final judicial disposition. Most participants had a history of previous incarceration, while only 18.4% were experiencing their first period of detention. Documented substance use was present in 54.2% of the sample.

At prison admission, 41.4% of participants had a documented psychiatric morbidity. Across the entire sample, Cluster B personality disorders were the most prevalent psychiatric diagnosis (30.6%), followed by mood disorders (17.5%) and psychotic disorders (2.7%). Overall, 41.3% of participants were receiving psychopharmacological treatment at the time of assessment.

### 3.2. Bivariate Analyses of Prisoners Stratified by Psychiatric Diagnosis on Admission

To examine demographic and clinical differences, the sample was stratified into two groups according to psychiatric status at admission (Table 2, Figure 1 and Figure 2). At prison admission, 41.4% had a psychiatric morbidity. Bivariate analyses showed that individuals with a psychiatric diagnosis at admission were significantly younger than those without a psychiatric diagnosis (39.3 ± 12.1 vs. 45.2 ± 13.0 years, *p* < 0.001), were more frequently of foreign nationality (χ^2^ = 31.641, *p* < 0.001), had a higher frequency of first incarceration (22.2% vs. 15.7%; χ^2^ = 4.807, *p* = 0.028), and more frequently reported substance use (84.6% vs. 32.6%; χ^2^ = 187.111, *p* < 0.001). No statistically significant differences were observed between the two groups with respect to legal status (*p* = 0.155).

On a clinical level, within the psychiatric-positive group, Cluster B personality disorders were the most prevalent condition (73.7%), followed by mood disorders (42.3%) and psychotic disorders (6.5%). Among mood disorders, cyclothymic disorder was documented in 20.5% of cases, bipolar disorder type II in 8.2%, and major depressive disorder in 5.5%. Strikingly, 99.6% of the subjects with a psychiatric diagnosis were actively receiving pharmacological treatment at the time of observation.

### 3.3. Multivariable Logistic Regression Analysis

A multivariable logistic regression model was fitted to identify factors independently associated with a psychiatric morbidity at prison admission (Table 3, Figure 3). The overall model was statistically significant compared to the null model (χ^2^ = 231.86, df = 8, *p* < 0.001), indicating a significant improvement in model fit. Goodness-of-fit indices demonstrated an adequate explanatory power, with McFadden’s R^2^ = 0.243, Cox & Snell’s R^2^ = 0.280, Nagelkerke’s R^2^ = 0.378, and Tjur’s R^2^ = 0.295.

After adjustment for all covariates, all substance use profiles were independently associated with substantially increased odds of a psychiatric diagnosis. The highest odds were observed among individuals reporting combined use of cannabis, cocaine, and psychostimulants, yielding a more than an elevenfold increase in risk compared with non-users (aOR = 11.70, 95% CI: 7.61–17.98, *p* < 0.001). Similarly, individuals reporting cannabis use only exhibited a highly comparable risk profile (aOR = 11.31, 95% CI: 4.06–31.52, *p* < 0.001). Combined cannabis and opioid use (aOR = 9.86, 95% CI: 4.64–20.94, *p* < 0.001) and opioid use only (aOR = 8.65, 95% CI: 3.18–23.54, *p* < 0.001) were also strongly and independently associated with psychiatric morbidity. Among sociodemographic factors, foreign nationality also remained a robust independent factor. Compared with Italian nationals, foreign individuals had significantly higher odds of presenting with a psychiatric diagnosis at admission (aOR = 4.39, 95% CI: 2.29–8.39, *p* < 0.001). Furthermore, legal status showed an association with psychiatric disorders at admission; compared with sentenced individuals, those awaiting final judicial disposition had 32% lower odds of having a psychiatric diagnosis at admission (aOR = 0.68, 95% CI: 0.47–1.00, *p* = 0.047).

By contrast, the association between age and psychiatric morbidity observed in the bivariate analysis was no longer statistically significant after multivariate adjustment (aOR = 0.99, *p* = 0.211), suggesting that the crude association was explained by confounding, primarily through the higher prevalence of substance use among younger individuals. First incarceration was not independently associated with psychiatric diagnosis at admission (aOR = 0.94, *p* = 0.791).

The results of the Firth sensitivity analysis were broadly consistent with those of the conventional model: the associations between the substance-use profiles and the outcome remained strong, the association with legal status was attenuated and no longer statistically significant, and age and first incarceration remained non-significant.

## 4. Discussion

The present study investigated the sociodemographic, legal, and clinical factors associated with psychiatric diagnosis at prison admission in a cohort of newly incarcerated individuals. The descriptive results highlight a sample characterized by a high proportion of psychiatric disorders. Overall, 41.4% of individuals had a documented psychiatric diagnosis, highlighting the substantial burden of mental health needs among individuals entering the correctional system [5,9,13,18,19] and reinforcing the importance of systematic mental health screening at prison admission.

Bivariate analyses showed significant differences between subjects with and without a psychiatric diagnosis for several clinical variables, including substance use, psychotic disorders, personality disorders, mood disorders, and pharmacological treatment. These findings are consistent with the literature emphasizing the high prevalence of psychiatric disorders and comorbidities in incarcerated settings [4,7,18,19,20].

Multivariate analyses identified independent factors associated with a psychiatric diagnosis on admission. The main finding of this study is the strong and independent association between substance use and a documented psychiatric diagnosis at admission. This association remained robust after adjustment for demographic and legal characteristics and was consistently observed across all substance use profiles. Compared with individuals without substance use, those reporting combined cannabis, cocaine, and psychostimulant use showed the highest odds of a documented psychiatric diagnosis (aOR = 11.70), followed by cannabis use alone (aOR = 11.31), combined cannabis and opioid use (aOR = 9.86), and opioid use alone (aOR = 8.65). These findings are consistent with the extensive literature describing the high prevalence of co-occurring substance use and psychiatric disorders among incarcerated populations [5,21].

In the Italian penitentiary framework, this clinical intertwining has reached critical proportions. Following the definitive closure of Forensic Psychiatric Hospitals and chronic community healthcare deficits, correctional facilities have *de facto* become primary repositories for severe psychiatric distress [16]. National data from Italy estimate that more than 40% of individuals experience mental health or psychological disorders during detention [19].

The coexistence of substance use disorders and psychiatric disorders represents one of the defining clinical characteristics of prison populations and reflects the complex interplay between biological vulnerability, adverse life experiences, trauma, and social disadvantage [5]. Psychiatric disorders and substance use frequently coexist through complex and bidirectional interplay: substance use may represent a maladaptive coping strategy for pre-existing psychiatric symptoms, while chronic substance exposure may contribute to the onset or exacerbation of psychiatric disorders [22,23]. However, the present study cannot establish the temporal sequence underlying this association. Given the retrospective cross-sectional design and the absence of individual interviews, our findings should be interpreted as associations rather than evidence of a causal relationship.

The particularly strong association observed among individuals reporting cannabis use alone deserves consideration. Rather than suggesting a direct causal relationship between cannabis use and psychiatric illness, this finding may indicate that cannabis use in this population represents a marker of broader psychosocial and psychiatric morbidity. This interpretation is supported by the high proportion of personality disorders (73.7%) and mood disorders (42.3%) among individuals with a documented psychiatric morbidity in our sample. Substance use should be considered within the broader framework of dual diagnosis in which psychiatric disorders and substance use mutually influence one another and require integrated clinical management [6]. Alternatively, cannabis use may coexist with other unmeasured risk factors that increase the likelihood of psychiatric disorders.

Foreign nationality emerged as an independent factor associated with a documented psychiatric diagnosis, with foreign individuals presenting more than fourfold higher odds of having a psychiatric diagnosis at entry prison compared with Italian nationals. This association remained stable after adjustment for all other covariates. This finding is consistent with previous evidence indicating that migrants and foreign nationals are frequently exposed to multiple psychosocial stressors, including migration-related trauma, social isolation, language barriers, and reduced access to mental healthcare prior to incarceration [24,25]. These factors may increase both the prevalence of mental disorders and the likelihood that psychiatric conditions remain untreated before incarceration. Therefore, prison admission may represent the first opportunity for comprehensive mental health assessment in this particularly vulnerable population.

Although prisoners with a psychiatric diagnosis were significantly younger in the unadjusted analyses, age was no longer associated with psychiatric diagnosis after adjustment for other covariates (*p* = 0.211). This finding suggests that the association between younger age and psychiatric diagnosis was largely explained by the higher prevalence of substance use among younger individuals rather than by age itself.

Previous studies have highlighted the close association between early substance use patterns, persistence of substance-related problems [26,27], and increased risk of psychiatric comorbidity, emphasizing the importance of early identification and intervention strategies [28,29]. It is important to note that our sample included only males: sex differences in substance use patterns and psychiatric morbidity have been reported, with males generally exhibiting higher prevalence of substance use disorders and different trajectories of substance-related problems compared with females [28].

By contrast, legal status became statistically significant only in the multivariable model. Compared with sentenced prisoners, individuals awaiting final judicial disposition had approximately one-third lower odds of having a psychiatric diagnosis at admission (aOR = 0.68). This finding should be interpreted cautiously given its borderline statistical significance, the multiple variables examined, and the observational design. Differences in the completeness or timing of clinical documentation according to legal status may represent one possible explanation, although this hypothesis could not be directly assessed in the present study. Sentenced prisoners may have experienced longer and more frequent contact with judicial, correctional, and healthcare services, increasing the probability that psychiatric disorders are formally recognized and documented. Conversely, psychiatric conditions among individuals entering prison while awaiting final judicial disposition may be less likely to have been previously identified, potentially leading to under-recognition of mental health needs during the early stages of detention because of the limited availability of previous clinical information. The association was attenuated and no longer statistically significant in the penalized model, consistent with the borderline nature of the finding in the conventional model.

### 4.1. Strengths and Limitations

The results of the present study should be interpreted considering several limitations. First, the cross-sectional and retrospective design precludes any inference regarding temporal or causal relationships between substance use and psychiatric disorders. Second, psychiatric status was determined from documented psychiatric diagnosis at admission, based on routine clinical assessment and available documentation rather than a standardized research diagnostic interview. The use of routinely collected clinical and administrative data represents a strength in terms of reflecting real-world correctional healthcare practice and allowing for the inclusion of consecutive admissions over the study period. However, these data were not originally collected specifically for research purposes. Therefore, previously diagnosed or documented psychiatric conditions may have been more readily identified than previously undiagnosed or incompletely documented psychiatric conditions. This may have resulted in underestimation of documented mental disorders at admission and limits the interpretation of our findings as estimates of the real prevalence of psychiatric disorders. Accordingly, the diagnostic categories reported in this study reflect clinical diagnoses and should not be interpreted as population-level prevalence estimates. Third, only male participants were included in the current study, a factor that may restrict the generalizability of these observations to female incarcerated populations. Finally, the study was conducted in a single correctional setting, which may limit the generalizability of the findings to other Italian prison systems.

Nonetheless, this study has several strengths. It included a relatively large cohort of newly admitted prisoners and simultaneously evaluated demographic, legal, and clinical factors associated with psychiatric morbidity. A particular strength of this work is its geographical and epidemiological focus, as it sheds light on a correctional facility in Southern Italy, a region historically underrepresented in the international forensic psychiatry literature. Given the unique socioeconomic context, regional welfare disparities, and specific migratory routes characterizing Southern European borders, providing reliable data from this specific territory offers a critical piece of the puzzle for understanding prison health dynamics beyond the standard data collected mostly from Northern metropolitan areas. Additionally, the analysis distinguished between different substance use profiles, allowing for a more detailed evaluation of their independent associations with psychiatric morbidity. The consistency of the multivariable findings across different model performance measures further supports the robustness of the results. Although the findings should not be assumed to be directly generalizable to all prison populations, the analytical approach and the clinical issues addressed are applicable beyond the Italian setting and may facilitate comparison with findings from other correctional systems.

### 4.2. Clinical Implications

The present findings have important implications for correctional mental healthcare. The high occurrence of psychiatric diagnosis at prison admission (~41%) and its strong association with substance use support the routine implementation of integrated mental health and addiction assessments for inmates. This structural demand is heavily reflected in our descriptive findings, which showed that virtually the entire psychiatric-positive cohort (~99%) was actively receiving psychopharmacological treatment upon entry. Nationally, the consumption of psychotropic drugs inside Italian prisons is estimated to be four to five times higher than in the general population [10], functioning as a primary mechanism to manage acute psychiatric symptoms, sleep deprivation, and substance withdrawal. Following the transfer of prison healthcare to the National Italian Health Service, significant regional disparities have emerged, leaving Southern facilities structurally vulnerable due to chronic shortages of dedicated staff, including forensic psychiatrists, psychologists, and cultural mediators. Early identification of individuals with co-occurring psychiatric and substance use disorders may facilitate timely multidisciplinary interventions, improve continuity of care, and reduce adverse clinical and behavioral health outcomes during detention, including psychiatric decompensation, withdrawal-related complications, self-harm, and institutional behavioral disturbances. Overall, prison admission represents a critical opportunity to identify individuals with complex mental health needs and to initiate integrated treatment strategies from the earliest stages of incarceration.

## 5. Conclusions

In conclusion, this study underscores a very high burden of psychiatric morbidity among newly incarcerated individuals in Southern Italy, demonstrating that clinical vulnerability is already deeply entrenched at the very threshold of the correctional system. Substance use—particularly polydrug use—and foreign nationality are the most prominent independent factors associated with a psychiatric diagnosis on admission. These findings provide a compelling, quantifiable portrait of the dual diagnosis crisis within a structurally challenged regional framework, where prisons frequently act as default repositories for neglected mental health needs.

Ultimately, these data demonstrate that admission to prisons cannot be treated as a mere administrative transition but should be operationalized as a critical public health window. Enhancing multidisciplinary resources and cross-institutional continuity of care is vital not only to mitigate institutional risks, psychiatric crises, and self-harm during early detention but also to break the cyclical pathways connecting mental illness, substance abuse, and criminal justice involvement.

## Figures and Tables

**Figure 1:**
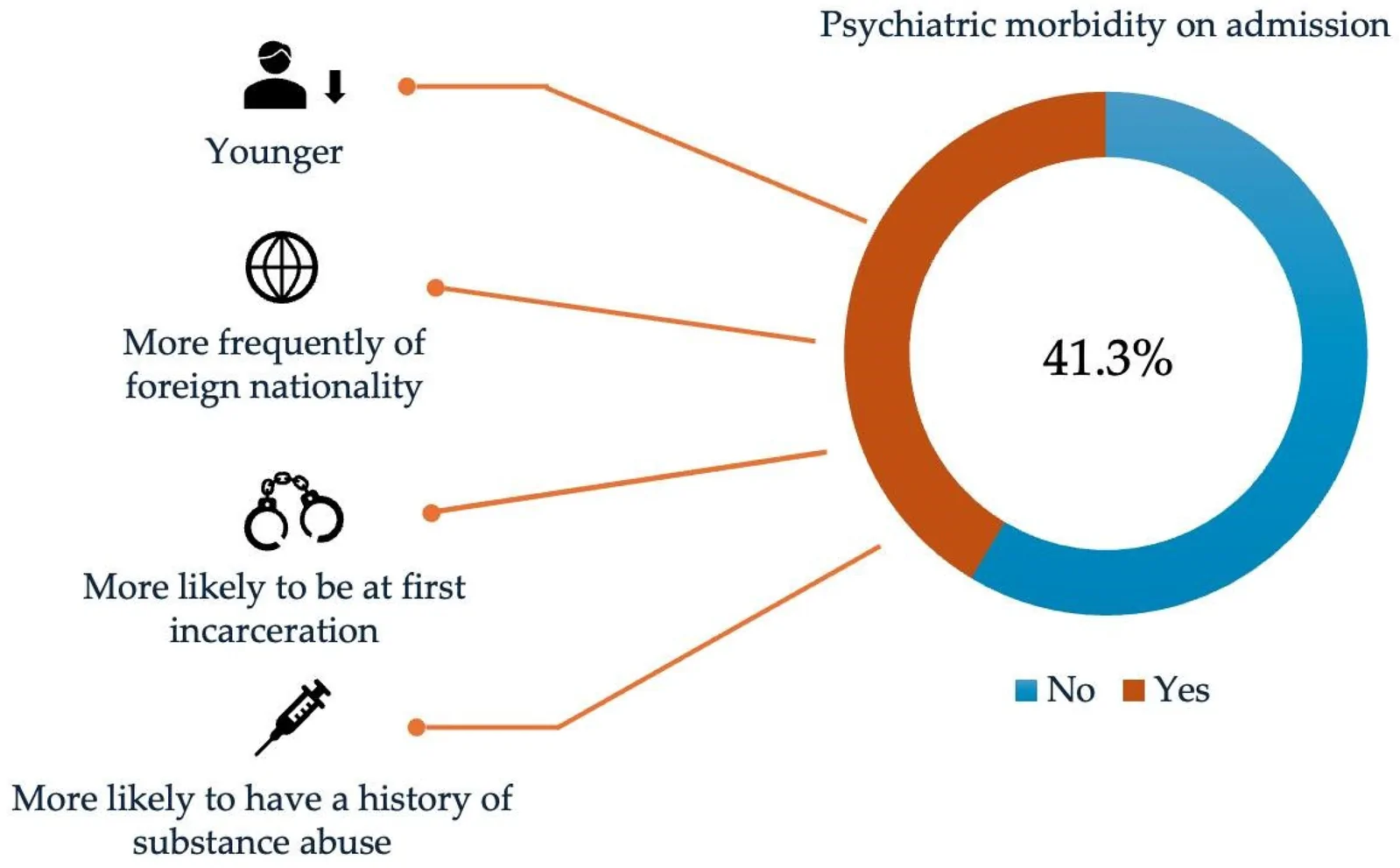
Characteristics of inmates with a psychiatric diagnosis on admission.

**Figure 2:**
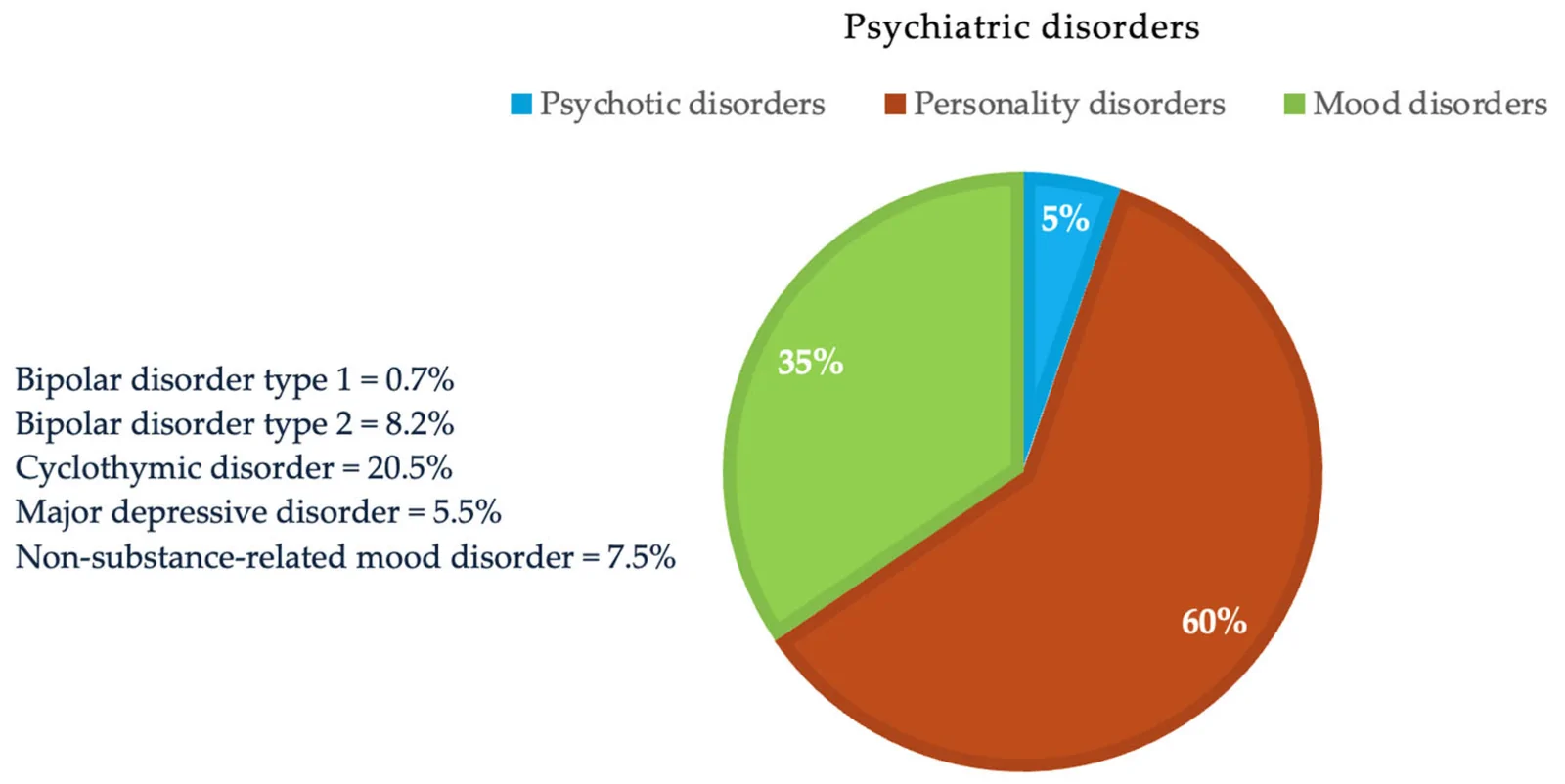
Proportion of psychiatric disorders among inmates with a psychiatric diagnosis.

**Figure 3:**
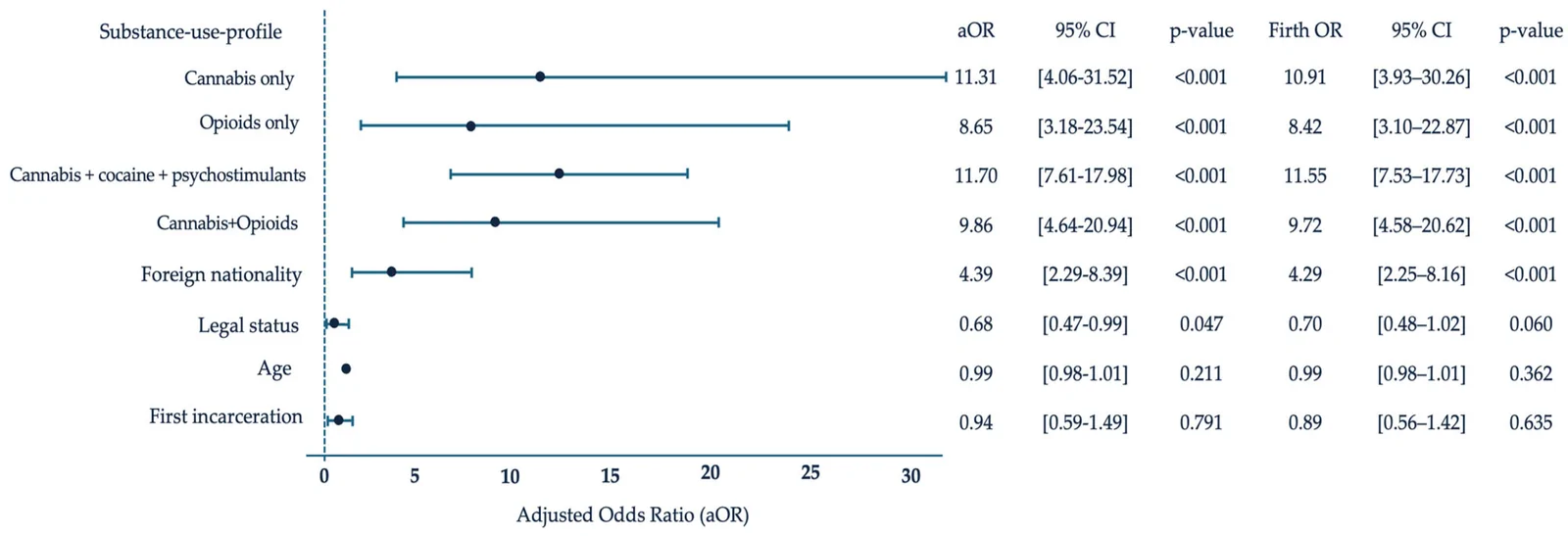
Independent factors associated with a psychiatric morbidity on admission.

**Table 1:** Socio-demographic, legal and clinical characteristics of the sample.

Variable		M	SD
Age	(years)	42.7	13.0
		Fr	%
Nationality	Italian	635	89.8
	Maghreb	26	3.7
	Other European countries	24	3.4
	Other	22	3.1
Legal status	Sentenced (final conviction)	263	37.2
	Awaiting final judicial disposition	444	62.8
First incarceration	YES	130	18.4
	NO	577	81.6
History of substance use	YES	383	54.2
	NO	324	45.8
Type of substance use	Cannabis only	19	2.7
	Opioids only	19	2.7
	Cannabis + cocaine + psychostimulants	306	43.3
	Cannabis + opioids	39	5.5
Psychiatric diagnosis on admission	YES	293	41.4
	NO	414	58.6
Psychotic disorders	YES	19	2.7
	NO	688	97.3
Personality disorders-Cluster B	YES	216	30.6
	NO	491	69.4
Mood disorders	YES	124	17.5
	NO	583	82.5
Type of mood disorder	Bipolar disorder type 1	2	0.3
	Bipolar disorder type 2	24	3.4
	Cyclothymic disorder	60	8.5
	Major depressive disorder	16	2.3
	Non-substance-related mood disorder	22	3.1
On pharmacological treatment	YES	292	41.3
	NO	415	58.7

Note: Data are expressed as Mean (M) and Standard deviation (SD) for continuous variables and as Frequency (Fr) and percentage (%) for categorical variables.

**Table 2:** Bivariate analyses of prisoners stratified by psychiatric morbidity on admission.

Variable		Negative *N* = 414	Positive *N* = 293	Statistics		Effect Size
		M (SD)	M (SD)	*t*	*p*	Cohen’s d
Age	(years)	45.2 (13.0)	39.3 (12.1)	6.476	<0.001	0.497
		Fr (%)	Fr (%)	χ^2^	*p*	Cramer’s V
Nationality	Italian	394 (95.2)	241 (82.2)	31.641	<0.001	0.212
	Maghreb	8 (1.9)	18 (6.1)			
	Other European countries	7 (1.7)	17 (5.8)			
	Other	5 (1.2)	17 (5.8)			
First incarceration	NO	349 (84.3)	228 (77.8)	4.807	0.028	0.082
	YES	65 (15.7)	65 (22.2)			
Legal status	Sentenced (final conviction)	145 (35.0)	118 (40.3)	2.023	0.155	0.053
	Awaiting final judicial disposition	269 (65.0)	175 (59.7)			
Substance use	NO	279 (67.4)	45 (15.4)	187.111	<0.001	0.514
	YES	135 (32.6)	248 (84.6)			
Substance-use profile	Cannabis only	7 (1.7)	12 (4.1)	188.236	<0.001	0.516
	Opioids only	8 (1.9)	11 (3.7)			
	Cannabis + cocaine + psychostimulants	104 (25.1)	202 (68.9)			
	Cannabis + opioids	16 (3.9)	23 (7.8)			

Note: Data are expressed as Mean (M) and Standard deviation (SD) for continuous variables and as Frequency (Fr) and percentage (%) for categorical variables. Cohen’s d was used as effect size measure for Student’s *t*-test, while Cramer’s V was used for Pearson’s Chi-squared (χ^2^) test.

**Table 3:** Multivariable logistic regression analysis of factors associated with psychiatric morbidity on admission.

Variable		β	SE	OR	95% CI	*p*	Firth OR	95% CI	*p*
Substance-use profile (Ref: Non-users)	Cannabis only	2.426	0.523	11.31	(4.06–31.52)	<0.001	10.91	(3.93–30.26)	<0.001
	Opioids only	2.158	0.511	8.65	(3.18–23.54)	<0.001	8.42	(3.10–22.87)	<0.001
	Cannabis + cocaine + psychostimulants	2.460	0.220	11.70	(7.61–17.98)	<0.001	11.55	(7.53–17.73)	<0.001
	Cannabis + opioids	2.288	0.384	9.86	(4.64–20.94)	<0.001	9.72	(4.58–20.62)	<0.001
Foreign Nationality (Ref: Italian)		1.480	0.330	4.39	(2.29–8.39)	<0.001	4.29	(2.25–8.16)	<0.001
Legal Status (Ref: Sentenced)		−0.386	0.194	0.68	(0.47–0.99)	0.047	0.70	(0.48–1.02)	0.060
Age		−0.010	0.008	0.99	(0.98–1.01)	0.211	0.99	(0.98–1.01)	0.362
First Incarceration (Ref: No)		−0.062	0.235	0.94	(0.59–1.49)	0.791	0.89	(0.56–1.42)	0.635

Notes: β = coefficient; SE = Standard Error; OR = Odds Ratio; CI = Confidence Interval; Ref = reference group. Sensitivity analysis using Firth penalized logistic regression yielded broadly consistent estimates.

## Data Availability

The data presented in this study are available on request from the corresponding author due to privacy and legal reasons.

## Abbreviations

The following abbreviations are used in this manuscript:

CI	Confidence interval
aOR	Adjusted odd ratio
DSM-5	Diagnostic and Statistical Manual of Mental Disorders-5
Fr	Frequency
M	Mean
OR	Odds Ratio
SD	Standard Deviation
WHO	World Health Organization
